# The iron-modulating hormone hepcidin is upregulated and associated with poor survival outcomes in renal clear cell carcinoma

**DOI:** 10.3389/fphar.2022.1080055

**Published:** 2022-12-02

**Authors:** Jian Huang, Wang Liu, Shiqi Song, Jean C. Li, Kaimei Gan, Chunxiao Shen, Jeffrey Holzbeierlein, Benyi Li

**Affiliations:** ^1^ Pathological Diagnosis and Research Center, The Affiliated Hospital of Guangdong Medical University, Zhanjiang, China; ^2^ Department of Urology, The University of Kansas Medical Center, Kansas, KS, United States

**Keywords:** HAMP, renal cancer, patient survival, disease progression, immune infiltration

## Abstract

**Background:** Reliable biomarkers are rare for renal cell carcinoma (RCC) treatment selection. We aimed to discover novel biomarkers for precision medicine. The iron-regulating hormone hepcidin (HAMP) was reportedly increased in RCC patient sera and tissues. However, its potential implication as a prognostic biomarker remains exclusive.

**Methods:** Multiple RNA-seq and cDNA microarray datasets were utilized to analyze gene expression profiles. Hepcidin protein expression was assessed using an ELISA assay in cell culture models. Comparisons of gene expression profiles and patient survival outcomes were conducted using the R package bioinformatics software.

**Results:** Five (HAMP, HBS, ISCA2, STEAP2, and STEAP3) out of 71 iron-modulating genes exhibited consistent changes along with tumor stage, lymph node invasion, distal metastasis, tumor cell grade, progression-free interval, overall survival, and disease-specific survival. Of which HAMP upregulation exerted as a superior factor (AUC = 0.911) over the other four genes in distinguishing ccRCC tissue from normal renal tissue. HAMP upregulation was tightly associated with its promoter hypomethylation and immune checkpoint factors (PDCD1, LAG3, TIGIT, and CTLA4). Interleukin-34 (IL34) treatment strongly enhanced hepcidin expression in renal cancer Caki-1 cells. Patients with higher levels of HAMP expression experienced worse survival outcomes.

**Conclusion:** These data suggest that HAMP upregulation is a potent prognostic factor of poor survival outcomes and a novel immunotherapeutic biomarker for ccRCC patients.

## Introduction

Renal cell carcinoma (RCC) is a malignant disease derived from the lining epithelium of tubules in the kidney and is the most common type of kidney cancer. There are three major types of RCC, clear cell (ccRCC), papillary (pRCC), and chromophobe (ChRCC). The ccRCC subtype is the most common one (about 75%), followed by pRCC (15%–20%) and ChRCC (5%) (Siegel et al., 2021). Some rare RCCs include collecting duct RCC, multilocular cystic RCC, medullary carcinoma, mucinous tubular and spindle cell carcinoma, and neuroblastoma-associated RCC. Based on the ClearCode34 model (Brooks et al., 2014), ccRCCs can be divided into two subtypes, namely good risk (ccA) and poor risk (ccB), which provides a prognostic stratification to improve risk assessment of recurrence and death for ccRCC patients. In addition, a recent comprehensive genomic, phenotypic and clinal analysis discovered several distinctive molecular and immune-related features in each RCC subtype, providing novel clues for developing subtype-specific therapies and management strategies (Ricketts et al., 2018). Despite all these efforts, the clinical success of risk stratification remains to be improved, and novel prognostic factors are awaiting individualized management, especially for immunotherapies (Rodriguez-Vida et al., 2016).

Over the past two decades, treatment options for RCC patients have changed from the first generation of immunotherapy with immunocytokines to targeted therapy with tyrosine kinase inhibitors (TKI), substantially improving patient survival outcomes (Albiges et al., 2019). Most recently, targeted immunotherapy with immune checkpoint inhibitors (ICI) emerged as an additive option to overcome kinase inhibitor resistance (Atkins et al., 2021). Innovative combinations of ICI or ICI plus TKI are now becoming the first line of the treatment strategy. The clinical response to these new combinational therapies is largely improved with prolonged survival (Rini et al., 2019). Successful immunotherapy depends on the therapeutic response from the immune effector cells within the tumor microenvironment, in other words, the tumor-infiltrating immune cells. Multiple immune cells form an ecosystem in the tumor tissue, and the infiltrating profiles are associated with therapeutic responsiveness and patient prognosis (Zhang et al., 2019). Identifying biomarkers strongly associated with these immune profiles in RCC patients is the key to making a precise decision for better treatment outcomes and improved survival. However, the limitations of the most studied biomarkers, like PD-L1, make necessary the identification of robust and novel biomarkers (Deleuze et al., 2020).

Iron is an essential nutrient involved in multiple cellular pathways. However, aberrant accumulation of labile iron is toxic to cells, causing oxidative stress and ferroptosis (Torti and Torti, 2020). Iron homeostasis, therefore, is tightly monitored at multiple levels, including iron uptake, storage, transportation, utilization, and secretion (Brown et al., 2020). There are more than 60 genes reported to modulate iron levels (Miller et al., 2011). Emerging evidence indicated that aberrant iron metabolism was involved in tumorigenesis and progression, including RCCs (Miller et al., 2011; Brown et al., 2020; Torti and Torti, 2020). In human RCC tissues, iron accumulation had long been reported (Dobrowolski et al., 2002), and the serum level of iron-binding protein Ferritin was considered a tumor biomarker for RCC patients (Essen et al., 1991; Kirkali et al., 1995). A recent study demonstrated the pivotal role of elevated labile iron levels in human RCC cells by promoting tumor cell proliferation and migration (Schnetz et al., 2020). Interestingly, sixteen iron regulatory genes were reported as a prognostic signature in breast cancer patients (Miller et al., 2011). However, in ccRCC patients, it is not clear which iron-modulating genes are tightly correlated with clinicopathological parameters and disease prognosis.

Hepcidin is a small peptide hormone encoded by the gene HAMP and is mainly produced by hepatocytes to regulate iron homeostasis (Krause et al., 2000; Park et al., 2001; Pigeon et al., 2001). Studies have demonstrated ectopic sources of hepcidin production, especially in several cancers derived from the kidney, colon, lung, breast, or prostate (Tesfay et al., 2015; Vela and Vela-Gaxha, 2018; Jerzak et al., 2020; Colorectal Cancer Cells, 2021; Fan et al., 2021; Schwartz et al., 2021). In RCCs, elevated serum levels of hepcidin peptides were first reported in 2009 (Kamai et al., 2009), and HAMP upregulation was confirmed by a recent study in RCC tissues (Traeger et al., 2019). A previous report showed that increased hepcidin expression in RCC tissues and patient sera correlated with disease progression, but the survival association was not significant, possibly due to a small cohort (Traeger et al., 2019). Interestingly, another recent report showed that HAMP expression was upregulated at the mRNA level in lung cancer tissues and was associated with immune infiltrations in the tumor microenvironment (Fan et al., 2021), indicating that HAMP might be a potential prognostic biomarker in cancers.

Therefore, we took a comprehensive approach and examined seventy-one iron-modulating genes for their correlation with clinicopathological parameters. Five out of 71 genes showed a significant correlation with all clinicopathological parameters in ccRCC patients. Among these five genes, HAMP upregulation exerted as a prime factor over the other four genes in distinguishing ccRCC and normal kidney tissues. HAMP upregulation was strongly correlated with four immune checkpoint protein genes, disease progression, and worse survival outcomes in ccRCC patients. These results indicate that HAMP upregulation might serve as a novel prognostic biomarker and immunotherapeutic target for ccRCC patients.

## Materials and methods

### Cell culture and reagents

Human ccRCC cell line Caki-1 (catalog #HTB-46) was obtained from ATCC (Manassas, VA). Cells were kept in McCoy’s 5A Modified Medium (ATCC Catalog #30-2007) supplied with 10% fetal bovine serum (FBS) and 1% penicillin/streptomycin at 37°C in a 5% CO_2_ setting. Tyrosine kinase inhibitor Sorafenib was purchased from Cayman Chemicals (Ann Arbor, MI) and dissolved in DMSO at 10 mM stock concentration. Bone morphogenetic proteins (BMP) and interleukin cytokines were synthesized by PeproTech (Cranbury, NJ) and dissolved in a 1% bovine serum albumin (BSA) carrier solution at a concentration of 10 mg/ml. The enzyme-linked immunoassay (ELISA) kit for the hepcidin assay (Catalog #EKF58061) was purchased from BioMatik (Wilmington, DE).

### Cell treatment and hepcidin ELISA assays

Caki-1 cells were seeded in a 6-well plate at a density of 4 × 10^4^ cells overnight and then treated with Sorafenib and cytokines for 24 h, as indicated in the figure. Cell culture media (conditioned media) were collected at the end of experiments and spanned for 3 min to remove cell debris. The supernatants were subjected to the ELISA assay for hepcidin analysis. The ELISA assay was performed using the assay protocol provided by the supplier.

### Gene expression analysis

Gene expression profiles were assessed at the mRNA levels in malignant and normal kidney tissues using the RNA sequencing (RNA-seq) dataset from the TCGA project (Cancer Genome Atlas Research, 2008) and the cDNA microarray dataset (Yusenko et al., 2009). For the RNA sequencing datasets, fragments per kilobase per million (FPKM) data were downloaded from the TCGA online portal and converted to the log_2_ value of Transcript Per Million reads (TPM) before statistical analysis. The RNA-seq data from 72 cases-matched normal and malignant kidney tissues were included for the pairwise comparisons. The cDNA microarray dataset was used to generate graphic images of grouped comparisons on the Oncomine™ platform (Rhodes et al., 2004). A ROC curve analysis compared HAMP expression levels in malignant and normal kidney tissues derived from ccRCC patients.

### Assessment of patient survival outcomes

Patient survival outcomes were assessed using the TCGA-KIRC dataset with the Kaplan-Meier curve approach (Nagy et al., 2021). A minimum *p*-value approach was utilized to determine the cutoff value of gene expression levels for splitting patients into high or low subgroups (Menyhart et al., 2018). Patient survival analysis was also conducted based on gender, ethnic race, tumor mutation burden, and enriched or decreased immune infiltration types in the tumor microenvironment.

### Gene correlation and enrichment evaluation

The correlation of gene expression was analyzed using the Spearman coefficient test. The correlation between HAMP expression and DNA methylation levels was analyzed using the TCGA-KIRC RNA-seq dataset (Cerami et al., 2012). The highly correlated genes (Spearman rho ≥ +/− 0.3) were subjected to gene enrichment analysis and visualization on the KOBAS platform (Wu et al., 2006). The significantly correlated genes were visualized using the R-package (version 3.6.3).

### Data presentation and statistical analysis

All quantitative data were presented as the MEAN with the SEM (standard error of the mean). Differences among multiple groups were analyzed using the Wilcoxon rank-sum test. Case-matched pair comparison was conducted using the Wilcoxon signed-rank test. The odds ratio was calculated using the binomial logistic regression analysis for HAMP expression on tumor progression. The differences in patient survival outcomes between the two subgroups were analyzed using the Kaplan-Meier survival plot. The hazard ratio (H.R.) was calculated using the Log-rank test (Menyhart et al., 2018). Each ROC curve was tested for effectiveness with the Youden index and compared relative potential with DeLong’s test.

## Results

### HAMP was identified as a superior factor in distinguishing ccRCC from benign tissues

We reasoned that a robust biomarker of prognostic factor and therapeutic target should be biologically relevant and tightly correlated with disease aggressiveness and patient survival outcome. With this notion in mind, we examined seventy-one iron-modulating genes as reported (Miller et al., 2011; Brown et al., 2020; Schnetz et al., 2020; Torti and Torti, 2020). Gene expression levels were compared between different groups of ccRCC patients stratified by tumor stage (early vs. late), lymph node invasion, distal metastasis, cancer grade (low vs. high), progression-free interval (PFI), overall survival (OS), and disease-specific survival (DSS). We looked for the genes that exhibited a strong and concordant alteration among all the clinicopathological parameters (Supplementary Figure S1). Among these 71 genes, five genes (HAMP, HMBS, ISCA2, STEAP2, and STEAP3) presented concordant alterations in all comparisons (Supplementary Table S1). Of these five genes, the expression levels of HAMP, HMBS, and STEAP3 genes were significantly increased along with disease progression and correlated with worse survival outcomes. In contrast, STEAP2 and ISCA2 gene expression was significantly decreased along with disease progression and correlated with favorite survival outcomes.

**FIGURE 1:**
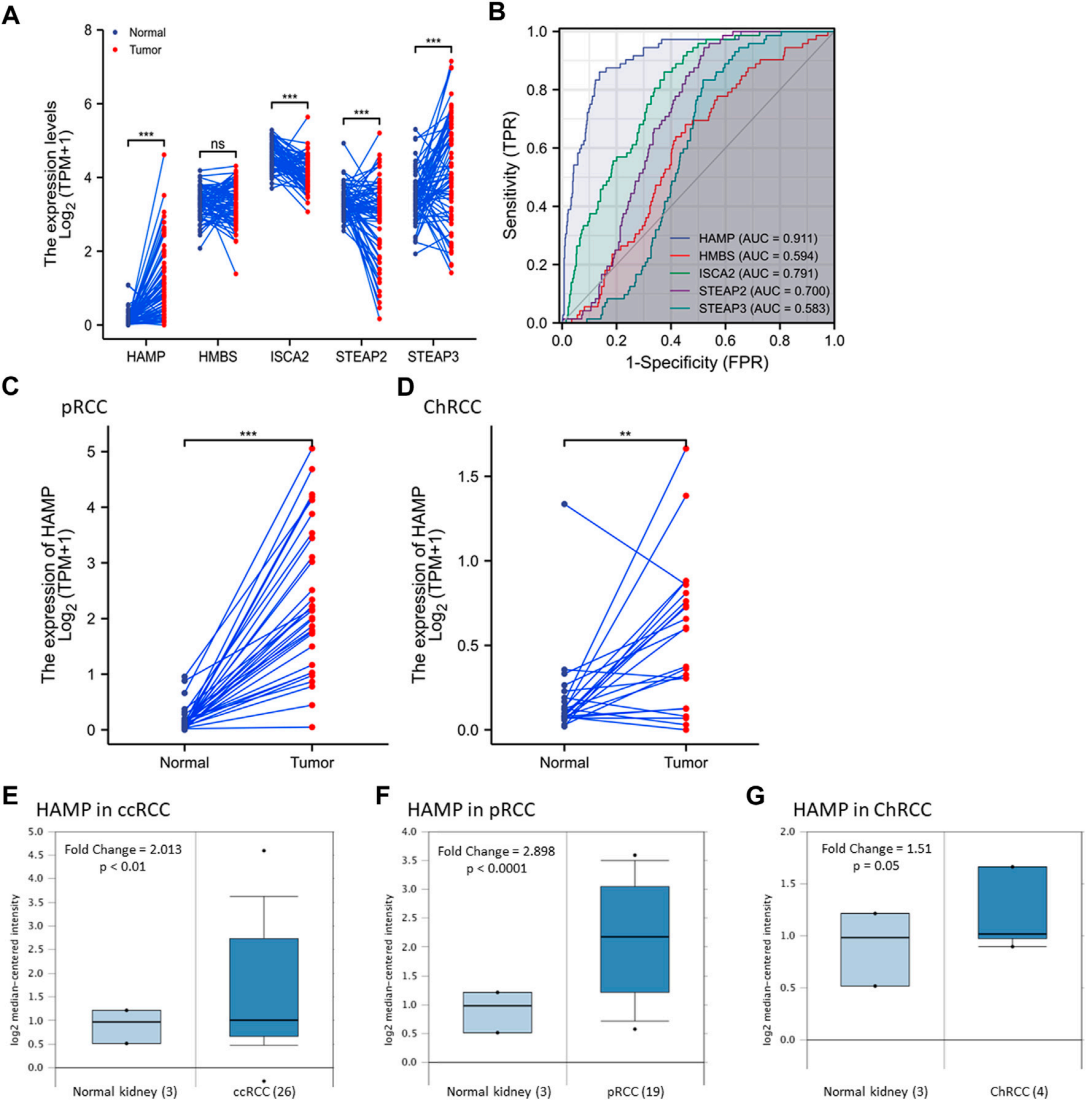
HAMP expression is upregulated in kidney cancers. **(A)** Gene expression was compared using the case-matched normal kidney and ccRCC tissues (TCGA -KIRC) from 72 cases. The *p*-values were derived from a paired *t*-test. **(B)** A Receiver Operating Characteristic (ROC) curve analysis for the diagnostic performance of iron-modulating gene expression in ccRCC tissues. AUC: Area under the ROC curve. **(C,D)** HAMP expression was compared using the case-matched normal kidney and ccRCC tissues (TCGA -KIRC) from 72 cases. The *p*-values were derived from a paired *t*-test. **(E–G)** HAMP expression levels were assessed using the cDNA microarray dataset as reported (Yusenko et al., 2009). The *p*-value was from a Student *t*-test. The asterisks indicate a significant difference between the two groups. **p < 0.01; *** *p* < 0.001; ns, no significance.

We examined gene expression alterations in a pairwise comparison of case-matched malignant and normal tissues derived from 72 patients in the TCGA-KIRC dataset. As shown in Figure 1A, the expression levels of four genes (HAMP, ISCA2, STEAP2, and STEAP3 but not HMBS) were significantly altered in malignant tissues compared to normal kidney tissues, of which HAMP gene expression displayed a consistent upregulation in all but one case. We then conducted a ROC analysis to determine their relative potentials in distinguishing malignant and normal kidney tissues. As shown in Figure 1B, HAMP expression was highly superior (AUC = 0.911, CI95: 0.879-0.944) over other four genes (AUC 0.594–0.791). These data indicated that HAMP upregulation is a potent diagnostic factor in ccRCC patients.

### HAMP expression is significantly upregulated in other RCC subtypes

We then analyzed HAMP expression in pRCC and ChRCC tissues with TCGA RNA-seq datasets. Our analysis revealed a significant increase of HAMP expression in pRCC and ChRCC tissues (Figures 1C,D). We then confirmed HAMP upregulation in ccRCC, pRCC, and ChRCC tissues (Figures 1E–G) with a secondary dataset derived from a cDNA microarray assay (Yusenko et al., 2009). In addition, HAMP upregulation was also evidenced in other human cancer types derived from the breast, colon, lung, head-neck, stomach, and esophagus (Supplementary Figure S2). However, HAMP was remarkably downregulated in human liver cancers, as described in our recent publication (Wang et al., 2021).

We also used a qualitative approach to analyze the associations of HAMP upregulation with clinicopathological parameters. Patients were divided into HAMP^high^ or HAMP^low^ groups based on the median level of HAMP expression (Table 1). In ccRCC patients, there were significantly more HAMP^high^ cases than HAMP^low^ cases in all clinicopathological parameters. Consistent with the functional role of HAMP in lowing circulating iron levels (Wojciechowska et al., 2021), HAMP^high^ group showed significantly more cases with lower hemoglobin levels than HAMP^low^ group. However, in pRCC and ChRCC patients, HAMP expression was not associated with these clinicopathological parameters (Supplementary Tables S2, S3). These results suggested that HAMP upregulation was tightly associated with clinicopathological parameters, in line with a recent report showing HAMP upregulation in chemical-induced rat kidney cancers (Matsumoto et al., 2017).

**TABLE 1:** Correlation of HAMP expression with c inicopathological parameters in ccRCC patients.

Characteristic	Low HAMP	High HAMP	*p*	Statistic	Method
n	269	270			
T stage, n (%)			**<0.001**	27.37	Chisq.test
T1	168 (31.2%)	110 (20.4%)			
T2	28 (5.2%)	43 (8%)			
T3	71 (13.2%)	108 (20%)			
T4	2 (0.4%)	9 (1.7%)			
N stage, n (%)			**0.040**	4.22	Chisq.test
N0	117 (45.5%)	124 (48.2%)			
N1	3 (1.2%)	13 (5.1%)			
M stage, n (%)			**0.002**	9.83	Chisq.test
M0	223 (44.1%)	205 (40.5%)			
M1	25 (4.9%)	53 (10.5%)			
Pathologic stage, n (%)			**<0.001**	30.74	Chisq.test
Stage I	167 (31.2%)	105 (19.6%)			
Stage II	24 (4.5%)	35 (6.5%)			
Stage III	51 (9.5%)	72 (13.4%)			
Stage IV	26 (4.9%)	56 (10.4%)			
Gender, n (%)			**0.013**	6.14	Chisq.test
Female	107 (19.9%)	79 (14.7%)			
Male	162 (30.1%)	191 (35.4%)			
Histologic grade, n (%)			**<0.001**	31.24	Chisq.test
G1	11 (2.1%)	3 (0.6%)			
G2	139 (26.2%)	96 (18.1%)			
G3	92 (17.3%)	115 (21.7%)			
G4	20 (3.8%)	55 (10.4%)			
Hemoglobin, n (%)			**0.003**		Fisher.test
Elevated	5 (1.1%)	0 (0%)			
Low	118 (25.7%)	145 (31.6%)			
Normal	107 (23.3%)	84 (18.3%)			
OS event, n (%)			**< 0.001**	19.35	Chisq.test
Alive	207 (38.4%)	159 (29.5%)			
Dead	62 (11.5%)	111 (20.6%)			
DSS event, n (%)			**< 0.001**	19.18	Chisq.test
Alive	230 (43.6%)	190 (36%)			
Dead	33 (6.2%)	75 (14.2%)			
PFI event, n (%)			**< 0.001**	12.59	Chisq.test
Alive	208 (38.6%)	170 (31.5%)			
Dead	61 (11.3%)	100 (18.6%)			
Age, median (IQR)	61 (52, 71)	60 (51, 69)	**0.350**	38004	Wilcoxon

### HAMP upregulation is associated with promoter DNA hypomethylation

**FIGURE 2:**
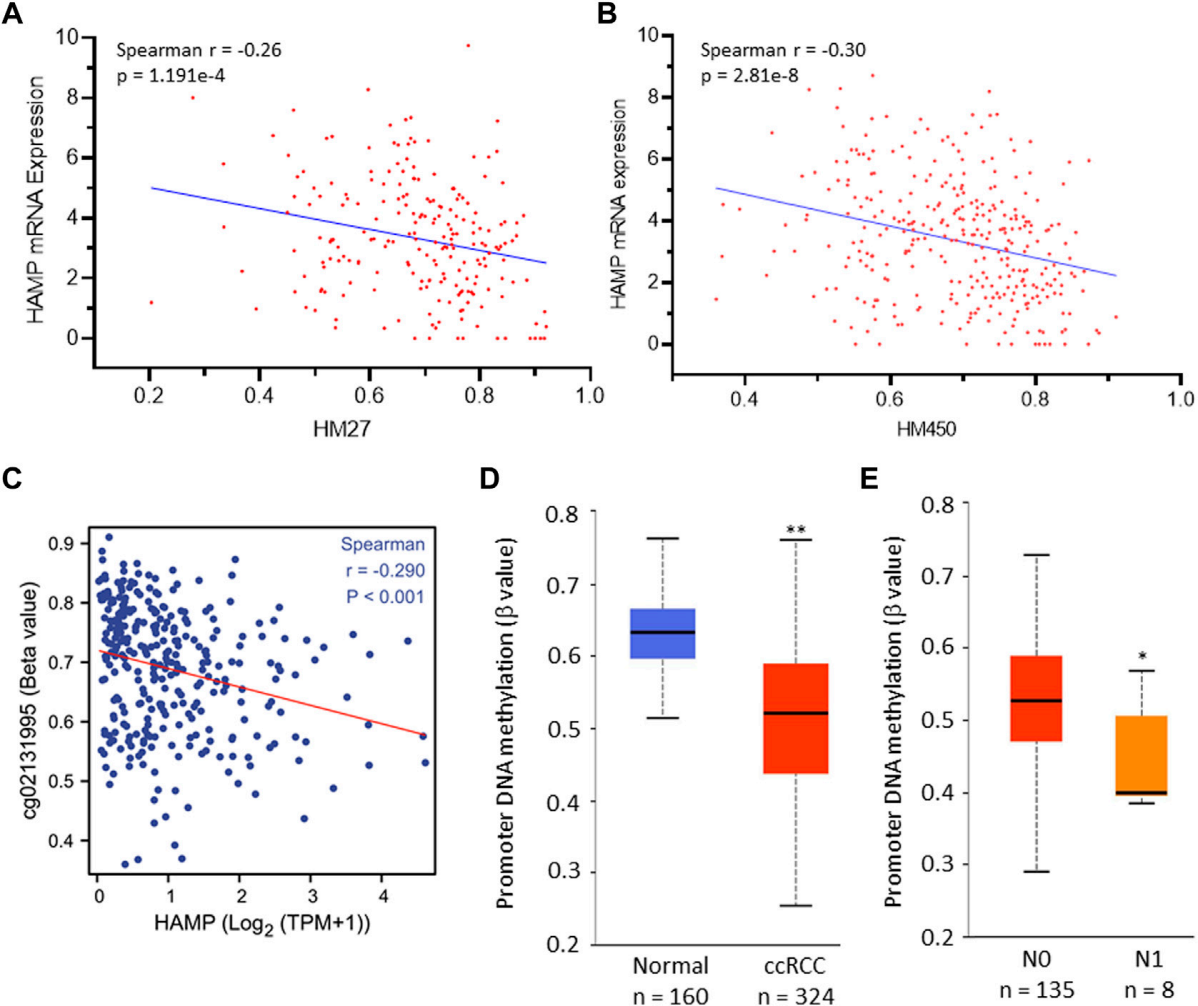
HAMP upregulation is correlated with HAMP gene hypomethylation in ccRCC tissues. **(A,B)** Spearman correlation coefficient was analyzed between HAMP gene promoter methylation and HAMP expression [RNA-seq V2 RSEM, log_2_ (value +1)] using the TCGA PanCancer RNA-seq dataset derived from Illumina Infinium Human Methylation BeadChips HM27 (217 ccRCC cases) and HM450 (319 ccRCC cases) platforms. **(C)** Spearman correlation coefficient was analyzed between HAMP gene methylation and HAMP expression. The probe cg02131995 covers the region of TSS+1297. TSS, transcription start site. **(D,E)** HAMP promoter methylation levels were compared quantitatively between normal kidney and ccRCC tumor tissues **(D)** and lymph node invasion status **(E)**. The asterisks indicate a statistical significance (Student *t*-test, * *p* < 0.05, ** *p* < 0.01).

Epigenetic modifications, such as promoter DNA methylation, are common mechanisms for transcriptional gene regulation (Angulo et al., 2021). We explored if HAMP upregulation was due to promoter demethylation. Our exploration found a significant correlation between HAMP mRNA expression and gene hypomethylation in two datasets derived from two different DNA methylation sequencing platforms, HM27 (Figure 2A) and HM450 (Figure 2B). Further analysis revealed a strong correlation between HAMP gene expression and DNA hypomethylation around the Exon-1/Intron-1 region (Figure 2C). Quantitatively, HAMP promoter methylation levels were significantly lower in ccRCC tissues than in normal kidney tissues (Figure 2D), which further decreased in tumors with lymph node invasion (Figure 2E). On the other hand, HAMP gene amplification was only found in one out of 751 (0.13%) ccRCC cases (Supplementary Figure S3). These data indicate that epigenetic modification (DNA hypomethylation) at the Exon-1/Intron-1 region, but not a structural/genetic abnormality, might be a potential mechanism of HAMP upregulation in ccRCC tumors.

### HAMP expression is upregulated by IL34 stimulation in ccRCC cells

**FIGURE 3:**
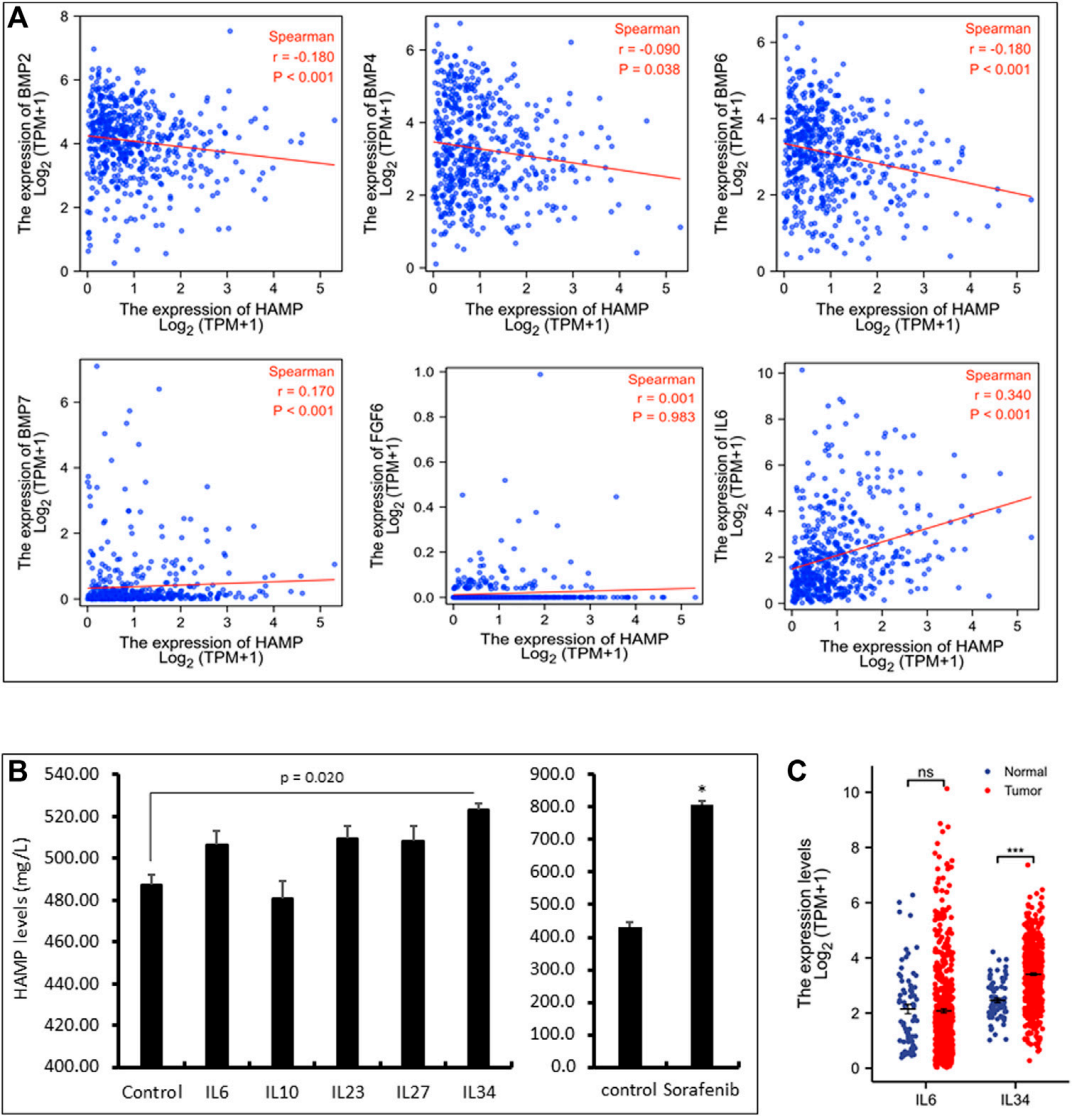
HAMP expression is positively correlated with IL6 and IL34 levels. **(A)** Spearman correlation analysis between HAMP and selective cytokines in ccRCC tissues was conducted using the TCGA-KIRC RNA-seq dataset (539 cases). **(B)** Caki-1 cells were treated with each cytokine (100 ng/ml) and Sorafenib (10 μM) for 24 h as indicated. Cell culture media were harvested for ELISA assay. The error bar represents the standard error of the MEAN (SEM). The asterisk indicates a significant difference from the control treatment (Student *t*-test, *p* < 0.05). **(C)** Comparison of IL6 or IL34 expression in ccRCC tissues compared to normal kidney tissues using the TCGA-KIRC RNA-seq dataset. Ns, no significance. *** *p* < 0.001 (paired *t*-test and Wilcoxon rank-sum test).

It has been reported that HAMP expression was stimulated by BMP proteins (BMP2, 4, 6, 7), inflammatory cytokine interleukin-6 (IL6), and fibroblast growth factor-6 (FGF6) (Vela and Vela-Gaxha, 2018). We analyzed their correlation with HAMP expression in ccRCC tumors. As shown in Figure 3A and Table 2, only IL6, but not BMP proteins and FGF6, were significantly correlated with HAMP expression in ccRCC tissues. To confirm the IL6 effect on HAMP expression, we treated ccRCC Caki-1 cells with IL6 and measured hepcidin production in the cell culture media. The kinase inhibitor Sorafenib was used as a positive control since it was reported to enhance HAMP expression (Mleczko-Sanecka et al., 2014). As shown in Figure 3B, Sorafenib treatment significantly increased hepcidin levels, as expected, whereas IL6 only had a weak but not significant enhancing effect on hepcidin levels, which was not in line with the correlation analysis data.

**TABLE 2:** Correlation of HAMP expression with selective cytokines in ccRCC tissues.

gene_name	gene_id	gene_biotype	cor_pearson	p_pearson	cor_spearman	p_spearman
IL6	ENSG00000136244	protein_coding	0.291928238247467	4.74759801918588E-12	0.341207966571258	3.66822271799337E-16
BMP2	ENSG00000125845	protein_coding	−0.137841529813564	0.001336256065816	−0.180268012425829	2.55380718683074E-05
BMP4	ENSG00000125378	protein_coding	−0.131051689300686	0.002298802285314	−0.089583684688162	0.037602488372864
BMP6	ENSG00000153162	protein_coding	−0.211059228879127	7.6355342805357E-07	−0.182787325933398	1.95620818941518E-05
BMP7	ENSG00000101144	protein_coding	0.050726704114415	0.239709573564412	0.17287086551273	5.47114433256835E-05
FGF6	ENSG00000111241	protein_coding	0.071491762963058	0.097307728627001	0.000944116692489	0.982553184080341
IL27	ENSG00000197272	protein_coding	0.489512371713915	7.95872392738277E-34	0.516262640971962	4.75676060579371E-38
IL23A	ENSG00000110944	protein_coding	0.423443221559579	7.3159630529362E-25	0.444824629820407	1.4970107605322E-27
IL10	ENSG00000136634	protein_coding	0.356362641173487	1.38856705887544E-17	0.422921707810165	8.46240818109115E-25
IL34	ENSG00000157368	protein_coding	0.301378925723637	8.83525701391414E-13	0.364201309893589	2.37945920425953E-18

We then conducted a whole-transcriptome correlation analysis with HAMP expression to explore other interleukin molecules that were associated with HAMP upregulation. Our analysis found that IL27, IL23A, IL10, and IL34 were strongly correlated with HAMP expression (Spearman coefficient r > 0.35, Table 2). We then examined their causative effects on hepcidin expression in Caki-1 cells. Surprisingly, only IL34 significantly stimulated hepcidin expression (Figure 3B). Further analysis revealed that IL34 but not IL6 gene expression was remarkably increased in ccRCC tissues (Figure 3C). These data suggested that IL34 is a potential driving factor for HAMP upregulation in ccRCC tissues.

### HAMP upregulation negatively impacts patient survival outcomes

**FIGURE 4:**
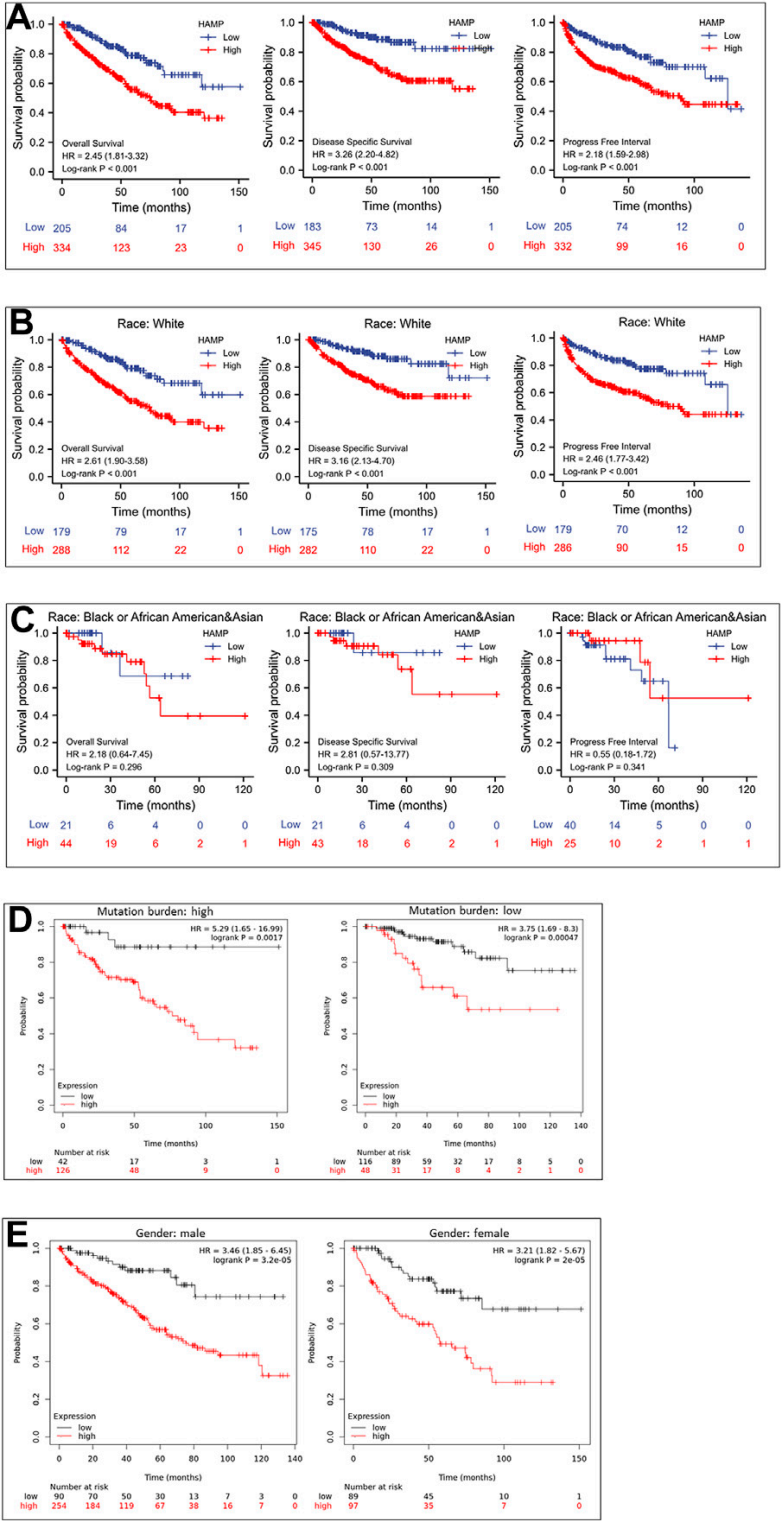
HAMP upregulation is associated with a poor survival outcome in ccRCC patients. **(A)** Patient survival outcomes of overall survival, disease-specific survival, and progression-free intervals were analyzed based on HAMP expression levels using the minimum *p*-value approach, as described (Menyhart et al., 2018). The 95% confidence interval of the H.R. value was listed within the parentheses. **(B–E)** Overall survival outcomes were compared between different groups using the minimum *p*-value approach described (Menyhart et al., 2018).

We then examined the association of HAMP upregulation with patient survival outcomes. As shown in Figure 4A, ccRCC patients with higher levels of HAMP expression had significantly worse survival outcomes (overall survival, disease-specific survival, and progression-free interval). This negative impact was statistically significant in the White race group with a statistical significance (Figure 4B) but not in the Black/Asian group of patients (Figure 4C). In patients with a higher tumor mutation burden (TMB), HAMP upregulation showed a substantial impact on overall survival than those with a lower TMB (HR 5.29 vs. 3.75, Figure 4D). However, the survival impacts were similar between male and female ccRCC patients (Figure 4E). These results demonstrated HAMP upregulation was a potent prognostic factor of unfavorable survival outcomes in the White race group of ccRCC patients with higher TMB levels.

**FIGURE 5:**
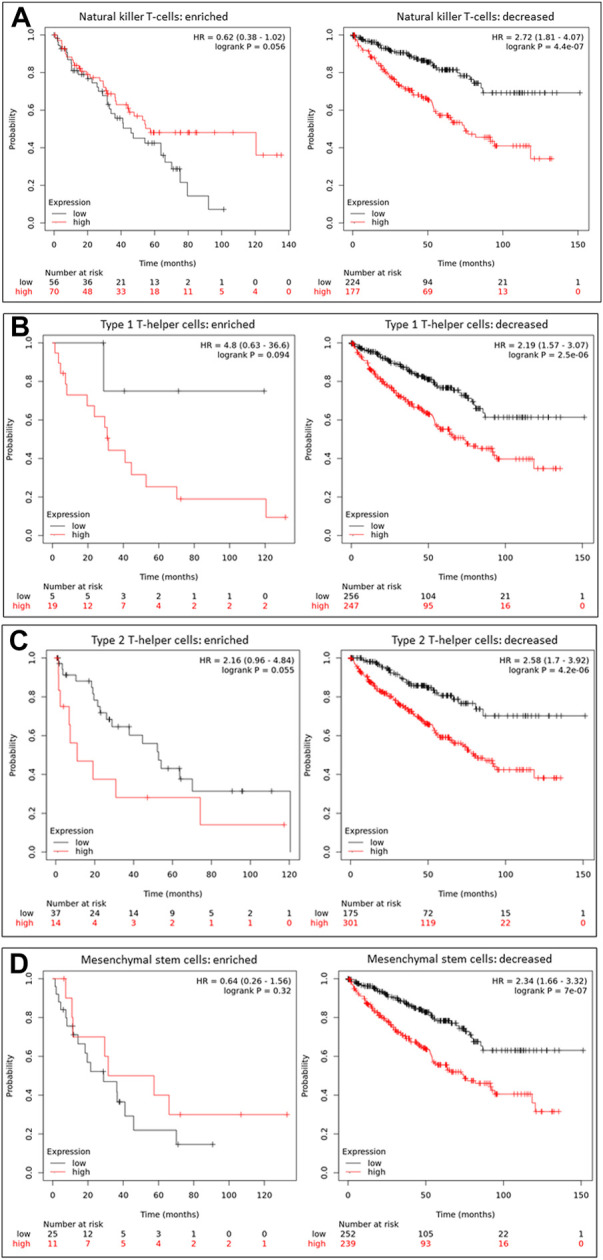
HAMP upregulation-related poor survival is diminished by enriched anti-cancer immune infiltration. Overall survival outcomes were analyzed based on HAMP expression levels using the minimum p-value approach on the K.M. Plotter platform, as described (Menyhart et al., 2018). Patients were stratified by enriched or decreased immune infiltrations, including NK cells **(A)**, Th1 cells **(B)**, Th2 cells **(C)**, and Mesenchymal stem cells **(D)**. The 95% confidence interval of the H.R. value was listed between the parentheses.

We compared patient survival outcomes based on immune infiltration enrichment to evaluate the clinical significance of immune infiltration expression concerning HAMP expression. Our analysis revealed that HAMP upregulation-associated worse survival outcomes were diminished in ccRCC patients who showed an intratumoral enrichment of natural killer (NK) cells, T Helper cells (T_H1/2_), and mesenchymal stem cells (Figure 5). Conversely, other immune infiltration enrichment, including eosinophils, macrophages, Treg cells, CD4^+^ memory cells, cytotoxic CD8^+^ T-cells, and B-cells, did not affect the HAMP upregulation-associated worse survival outcomes (Supplementary Figure S4). These results suggest that HAMP upregulation-related negative impact on patient survival only occurred when the tumor immune microenvironment was weakened due to reduced infiltration of anti-cancer immune cells.

### HAMP upregulation is associated with immune checkpoint genes

We next conducted a gene-set enrichment analysis (GSEA) to determine the most relevant pathways correlated with HAMP expression. Based on the whole-transcriptome correlation analysis on the TCGA-KIRC dataset, 1725 genes were positively correlated with HAMP expression, while 1188 genes were negatively correlated with HAMP expression based on a Spearman r cutoff value ± 0.3. These genes were then subjected to the GSEA analysis. Our results showed that the most significantly enriched clusters/pathways were immune regulatory and inflammatory cytokine/chemokines, as shown in Table 3 and Supplementary Figure S5.

**TABLE 3:** GSEA summary of HAMP-related gene pathways in ccRCC tissues.

ID	Set Size	enrichmentScore	NES	P-value	p.adjust	Q-value
HALLMARKINFLAMMATORY_RESPONSE	199	0.650427409	2.42527	0.001767	0.0068157	0.0031567
HALLMARK_INTERFERON_GAMMA_RESPONSE	200	0.6475499	2.414132	0.001776	0.0068157	0.0031567
HALLMARK_EPITHELIAL_MESENCHYMAL TRANSITION	200	0.637416355	2.376353	0.001776	0.0068157	0.0031567
HALLMARK_IL6 JAK_STAT3_SIGNALING	87	0.666791889	2.223887	0.001887	0.0068157	0.0031567
HALLMARK_COMPLEMENT	200	0.5761503	2.147947	0.001776	0.0068157	0.0031567
HALLMARK_INTERFERON_ALPHA_RESPONSE	97	0.610881595	2.068789	0.001908	0.0068157	0.0031567
HALLMARK E2F TARGETS	200	0.541685168	2.019457	0.001776	0.0068157	0.0031567

**FIGURE 6:**
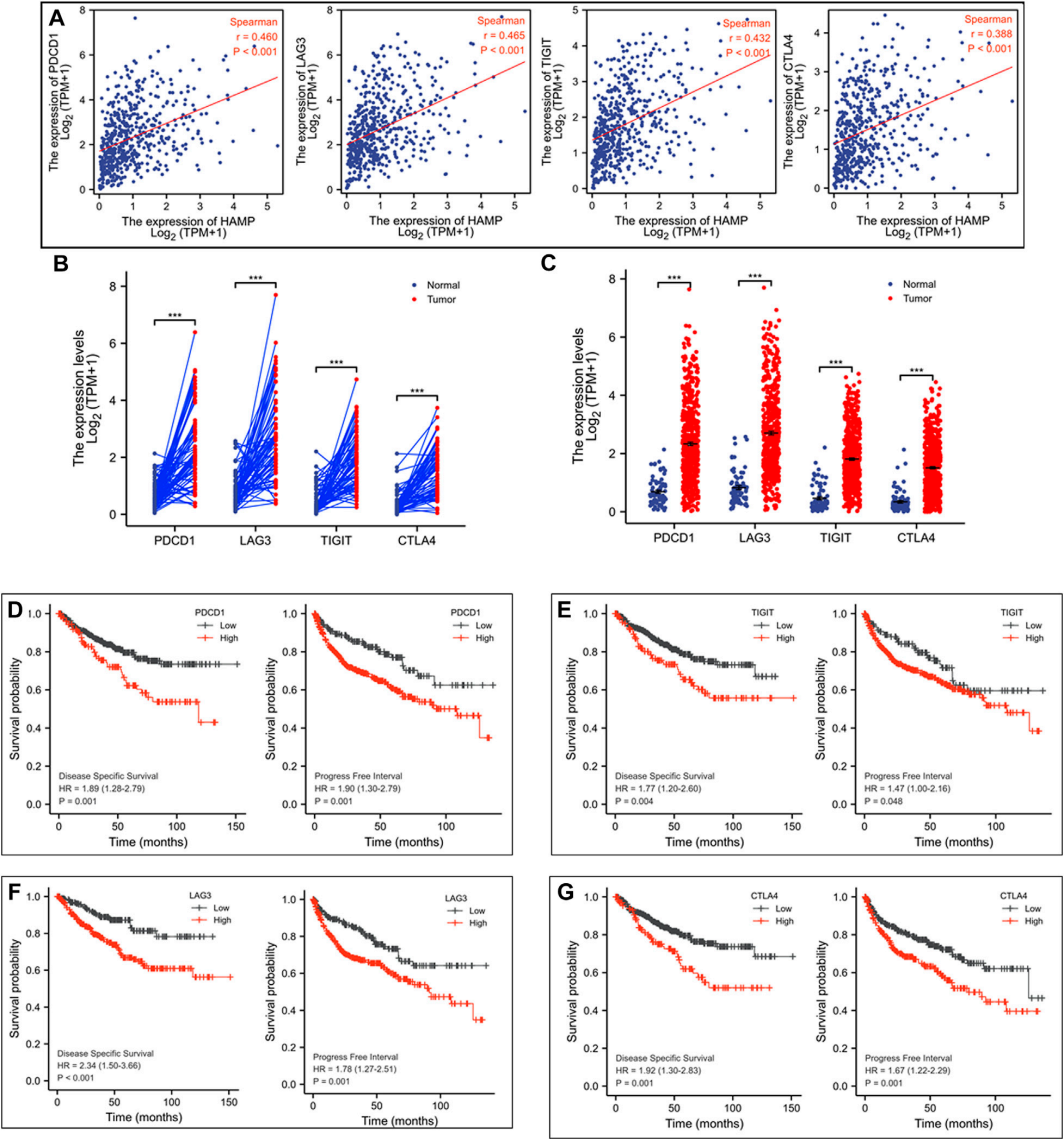
HAMP expression is associated with immune checkpoint receptors in ccRCC tissues. **(A)** Spearman correlation was analyzed between HAMP expression and immune checkpoint genes, and a strong correlation (Spearman r > 0.35) was noticed between HAMP and PDCD1, LAG3, TIGIT, and CTLA4. **(B–C)** The expression of the immune checkpoint genes was analyzed using the TCGA-KIRC dataset in a case-matched pairwise comparison (panel **B**) or group cohort (panel **C**) The *p*-values were derived from a paired *t*-test or Wilcoxon rank-sum test. **(D–G)** Patient survival outcomes of disease-specific and progression-free intervals were analyzed based on individual genes using the Kaplan-Meier curve, as described (Liu et al., 2018). The 95% confidence interval of the H.R. value was listed between the parentheses. The *p*-values were derived from the Log-rank test.

We then focused our analysis on immune-related genes (Huang et al., 2019; Simonaggio et al., 2021). A strong correlation (Spearman r > 0.35) was identified between HAMP expression and four major inhibitory checkpoint genes, PDCD1, LAG3, TIGIT, and CTLA4 (Figure 6A). In parallel with HAMP upregulation, these four checkpoint genes were also significantly upregulated in ccRCC tissues compared to normal kidney tissues, either in case-match pairwise (Figure 6B) or group cohort comparison (Figure 6C). Their upregulations were also associated with a significantly worse disease-specific survival and progression-free interval (Figures 6D–G), which were in line with a recent report (Zhang et al., 2019). These results indicate that HAMP and immune checkpoint genes (PDCD1, LAG3, TIGIT, and CTLA4) were coordinately upregulated in ccRCC tissues with a strong clinical impact on patient survival outcomes.

## Discussion

In this study, we found that HAMP expression was significantly upregulated and consistently correlated with clinicopathological parameters in ccRCC patients, including cancer grade, tumor stage, lymph node metastasis, distal metastasis, molecularly classified subgroups, disease progression, and patient survival. HAMP expression was positively associated with major immune checkpoint factors (PDCD1/LAG3/TIGIT/CTLA4). These immune checkpoint genes were also significantly upregulated in ccRCC tissues. They negatively impacted patient survival in parallel with HAMP upregulation. The adverse effects of HAMP upregulation on patient survival were diminished in an anti-cancer-enriched immune microenvironment. These results demonstrated that HAMP upregulation is a novel prognostic factor in disease progression and survival outcomes and a therapeutic target of immunotherapy for ccRCC patients.

Hepcidin protein levels in the sera and HAMP mRNA expression in tumor tissues were first reported more than 10 years ago in a small cohort (*n* = 32) of metastatic ccRCC patients (Kamai et al., 2009), which showed a positive correlation of HAMP expression in serum or tumor tissue with cancer grade, tumor stage, distance metastasis, and serum IL-6 levels. A recent report with a relatively larger cohort of ccRCC patients (*n* = 94) also consistently showed an increased serum hepcidin level in ccRCC patients compared to non-genitourinary or non-anemic patients (Traeger et al., 2019). High serum hepcidin levels were also significantly associated with distal metastasis and disease progression but not patient survival outcomes. In line with these two reports, our results from a large cohort confirmed the strong correlation of HAMP upregulation with cancer grade, tumor stage, lymph node metastasis, distal metastasis, disease progression, and patient survival. Specifically, HAMP upregulation in ccRCC tissues had an odds ratio of 4.089 for lymph node invasion and 2.306 for distal metastasis. The hazard ratio of HAMP upregulation for disease-specific survival was 3.26, and disease progression was 2.18. The main difference between our study and the previous two reports (Kamai et al., 2009; Traeger et al., 2019) was the case numbers (523 vs. 32 or 94), which provide substantial statistical power for survival analysis. Therefore, our results demonstrated that HAMP upregulation in ccRCC tissues is a decisive risk factor for rapid disease progression and poor survival outcomes.

Hepcidin is the primary hormone regulating iron homeostasis by controlling iron efflux from enterocytes, hepatocytes, and macrophages (Torti and Torti, 2020). It has been well documented that HAMP expression is positively regulated by BMP2/4/6/7 and IL6 (Vela and Vela-Gaxha, 2018), while tumor-derived IL6 suppresses T_H1_ cell functionality (Jones and Jenkins, 2018). In addition, tumor-derived BMP4 was shown to induce M2 macrophage polarization associated with tumor progression (Martinez et al., 2017). However, our analysis showed a negative correlation of BMP2/4/6 with HAMP expression in ccRCC tissues. Treatment of ccRCC Caki-1 cells with BMP2/6/7 proteins had no stimulatory effect on hepcidin levels. Although IL6 expression was strongly correlated with HAMP expression, it had no significantly stimulatory effect on hepcidin expression in Caki-1 cells. Surprisingly, IL34, a newly identified ligand for the colony-stimulating factor 1 (CSF1) receptor (Lin et al., 2008), significantly stimulated hepcidin expression. It has been shown that tumor cell-derived IL34 was implicated in therapy resistance and disease progression (Otsuka et al., 2021). Especially, tumor cell-derived IL-34 reduced immunotherapy efficacy by modulating myeloid cell activity (Hama et al., 2020). A further mechanistic investigation is underway by our group to elucidate the functional role of IL34 in HAMP expression and tumor progression.

Immune infiltration in the tumor microenvironment is crucial in patient response to various treatments (Zhang and Zhang, 2020). A better understanding of the immune infiltration concerning specific gene expression patterns will provide critical parameters in determining an immune precision medicine for cancer patients. In this study, our data revealed that HAMP upregulation-dependent negative impact on overall survival was diminished in an anti-cancer immune cell-enriched environment. In addition, four immune checkpoint genes (PD-1, LAG3, TIGIT, and CTLA4) were strongly correlated with HAMP upregulation, significantly upregulated in ccRCC tissues, and negatively impacted patient survival outcomes. These results provided a valuable clue for further investigation into the role of HAMP in the immune escape mechanism.

## Conclusion

This study demonstrated that HAMP upregulation was associated with clinicopathological parameters in ccRCC patients. HAMP upregulation had a substantial impact on disease progression and patient survival outcomes. Most importantly, HAMP upregulation was highly associated with increased expression of immune checkpoint factors in ccRCC tissues. Therefore, HAMP upregulation may serve as a novel prognostic factor and a therapeutic factor.

## Data Availability

The datasets presented in this study can be found in online repositories. The names of the repository/repositories and accession number(s) can be found in the article/Supplementary Material.

## Supplementary material

Supplementary Figure S1 Schematic Workflow of the study design.

Supplementary Figure S2 HAMP alteration in Pan-Cancer comparison. HAMP expression in indicated tumor tissues was compared to corresponding normal tissues using the TCGA datasets. The asterisk indicates a significant difference by Wilcoxon rank-sum test (* *p* < 0.05, ** *p* < 0.01; *** *p* < 0.001). The p-values were derived from the comparison between indicated groups.

Supplementary Figure S3 Structural alterations of HAMP gene in ccRCC tissues. Data were derived from 4 studies from 820 samples of 751 patients. The figure panel was downloaded from the cBioportal platform.

Supplementary Figure S4 HAMP upregulation-related poor survival is not affected by selective immune enrichment. Overall survival outcomes were analyzed based on HAMP expression levels using the minimum p-value approach on the K.M. Plotter platform, as described (32). Patients were stratified by enriched or decreased immune infiltrations as indicated. The 95% confidence interval of the H.R. value was listed between the parentheses.

Supplementary Figure S5 A representative GSEA curve for HAMP-related gene enrichment.

Supplementary Table S1 Relationship of iron-modulating genes with clinicopathological parameters in ccRCC patients. Footnotes: Gene expression comparisons between indicated groups were conducted using the Wilcoxon rank-sum test (* *p* < 0.05, ** *p* < 0.01; *** *p* < 0.001). Red font denotes upregulation; green denotes downregulation; ns stands for no significance.

Supplementary Table S2 Relationship of HAMP expression with clinicopathological parameters in pRCC patients.

Supplementary Table S3 Relationship of HAMP expression with clinicopathological parameters in ChRCC patients.

## Abbreviations

AUC, Area Under the ROC curve; ATCC, American Tissue Culture Collection; BMP, Bone morphogenetic proteins; BMPER, BMP binding endothelial regulator; ccRCC, clear cell RCC; ChRCC, chromophobe RCC; ELISA, enzyme-linked immunoassay; ERFE, erythroferrone; FPKM, fragments per kilobase per million; GSEA, gene set enrichment analysis; HAMP, HAMP gene; HJV, hemojuvelin; H.R., hazard ratio; KIRC, Kidney Renal Clear Cell Carcinoma; IL6, interleukin 6; N.K., natural killer cell; pRCC, papillary RCC; RCC, renal cell carcinoma; RNA-seq, RNA sequencing; RSEM, RNA-Seq by Expectation-Maximization; SEM, standard error of the mean; SOSTDC1, sclerostin domain containing 1; TCGA, The Cancer Genome Atlas; TME, tumor microenvironment; TPM, Transcript Per Million reads; TMPRSS6, transmembrane serine protease 6; Th17, T helper 17 cell; Treg, regulatory T-cells; TSS, transcription start site.
