# Supplementary figures and images for: The iron-modulating hormone hepcidin is upregulated and associated with poor survival outcomes in renal clear cell carcinoma
Source: Front Pharmacol. 2022 Dec 2;13:1080055. doi: 10.3389/fphar.2022.1080055 (PMC9757070; doi:10.3389/fphar.2022.1080055)

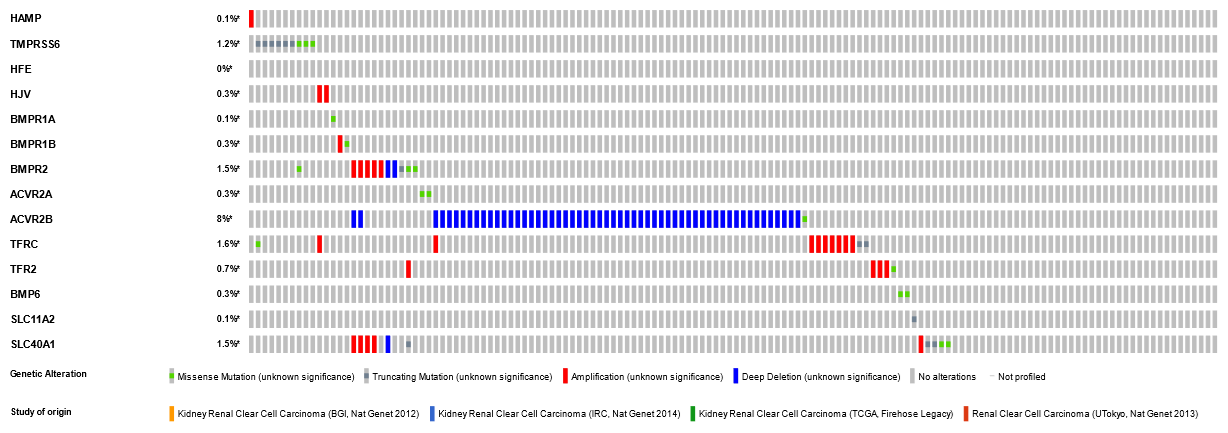

Supplement: Supplementary file 3 [file Image3.TIF]

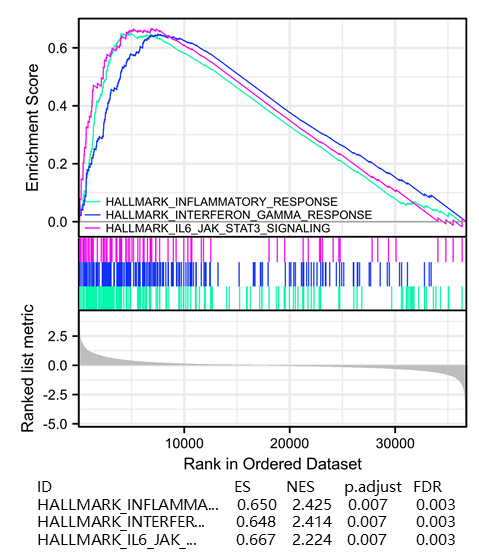

Supplement: Supplementary file 4 [file Image4.TIF]

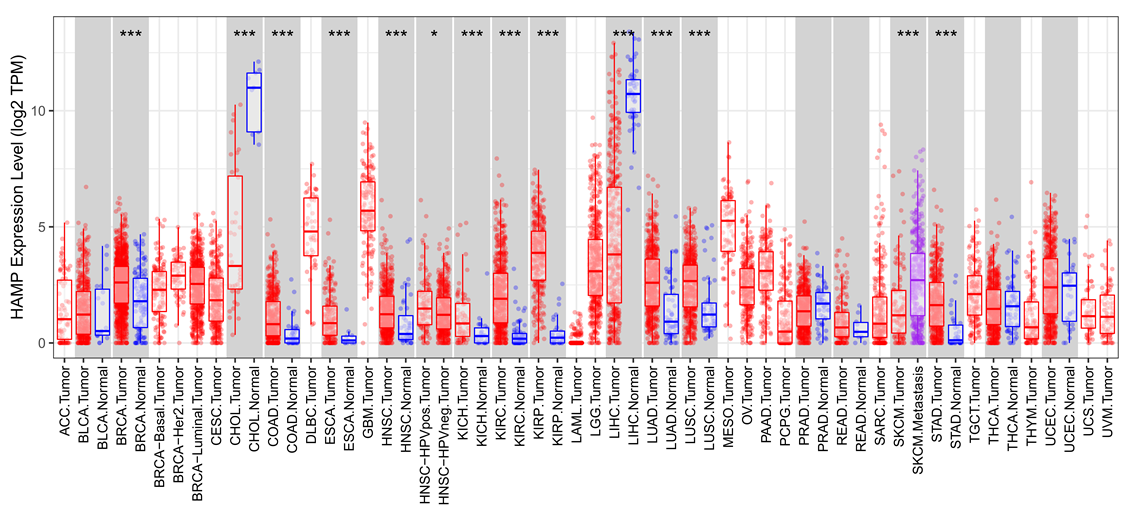

Supplement: Supplementary file 5 [file Image2.TIF]

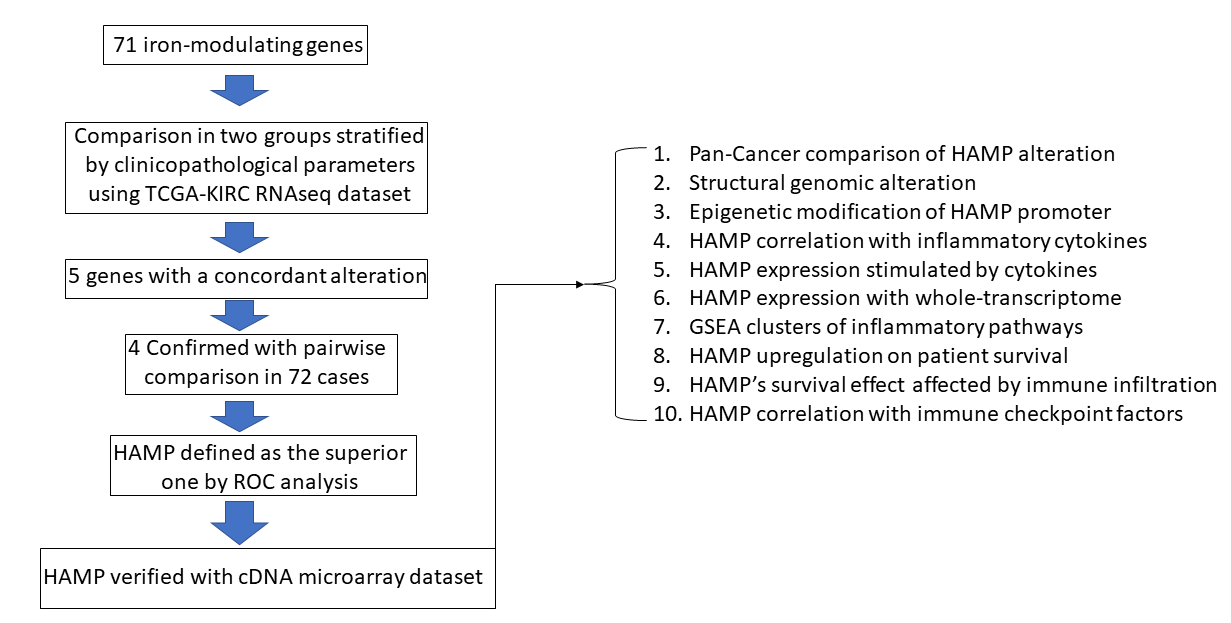

Supplement: Supplementary file 6 [file Image1.TIF]

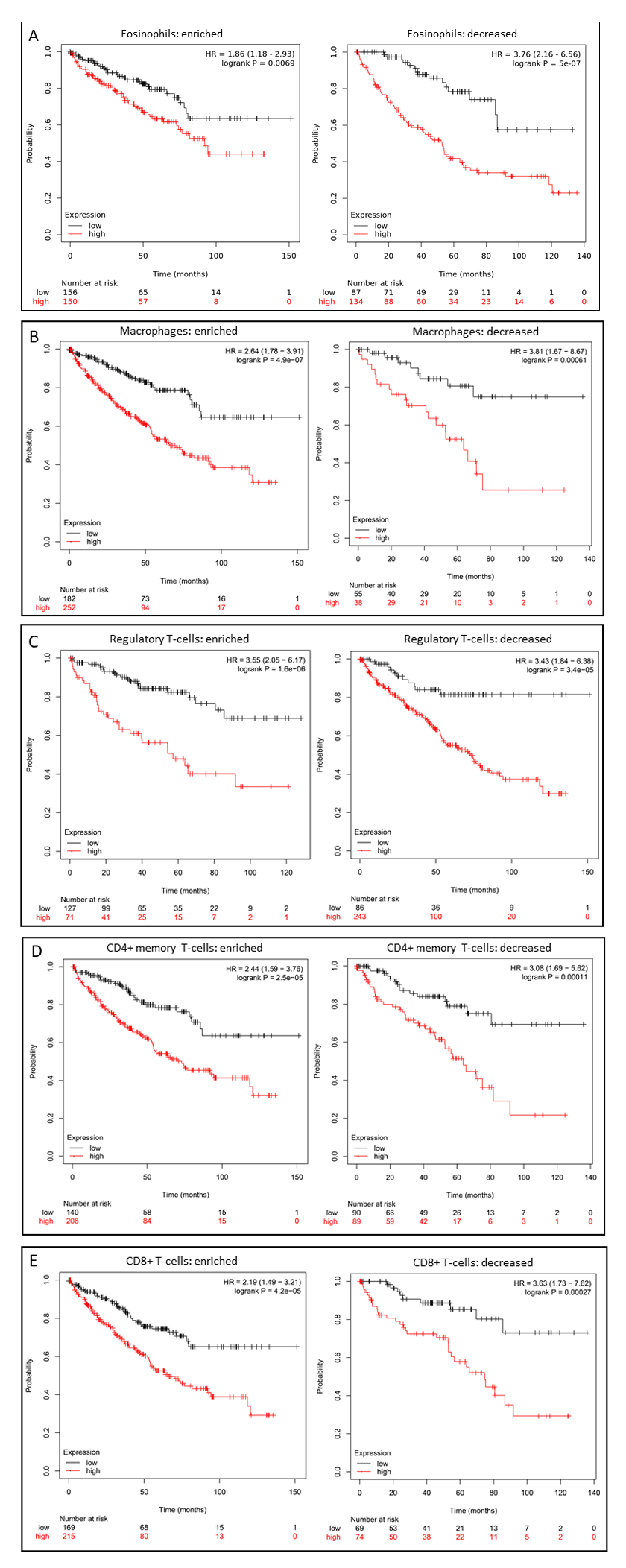

Supplement: Supplementary file 8 [file Image5.TIF]
